# Long-term opioid use following bicycle trauma: a register-based cohort study

**DOI:** 10.1007/s00068-022-02103-w

**Published:** 2022-09-12

**Authors:** Evelyne Zibung, Erik von Oelreich, Jesper Eriksson, Christian Buchli, Caroline Nordenvall, Anders Oldner

**Affiliations:** 1 Department of Molecular Medicine and Surgery, Karolinska Institutet, Stockholm, Sweden; 2 Section of Anesthesiology and Intensive Care Medicine, Department of Physiology and Pharmacology, Karolinska Institutet, Stockholm, Sweden; 3 Perioperative Medicine and Intensive Care, Karolinska University Hospital, Stockholm, Sweden; 4 Colorectal Surgery Unit, Department of Pelvic Cancer, Karolinska University Hospital, Stockholm, Sweden

**Keywords:** Bicycle trauma, Prescription opioids, Long-term opioid use, Opioid crisis

## Abstract

**Purpose:**

Chronic opioid use is a significant public health burden. Orthopaedic trauma is one of the main indications for opioid prescription. We aimed to assess the risk for long-term opioid use in a healthy patient cohort.

**Methods:**

In this matched cohort study, bicycle trauma patients from a Swedish Level-I-Trauma Centre in 2006–2015 were matched with comparators on age, sex, and municipality. Information about dispensed opioids 6 months prior until 18 months following the trauma, data on injuries, comorbidity, and socioeconomic factors were received from national registers. Among bicycle trauma patients, the associations between two exposures (educational level and injury to the lower extremities) and the risk of long-term opioid use (> 3 months after the trauma) were assessed in multivariable logistic regression models.

**Results:**

Of 907 bicycle trauma patients, 419 (46%) received opioid prescriptions, whereof 74 (8%) became long-term users. In the first quarter after trauma, the mean opioid use was significantly higher in the trauma patients than in the comparators (253.2 mg vs 35.1 mg, *p* < 0.001) and fell thereafter to the same level as in the comparators. Severe injury to the lower extremities was associated with an increased risk of long-term opioid use [OR 4.88 (95% CI 2.34–10.15)], whereas high educational level had a protecting effect [OR 0.42 (95% CI 0.20–0.88)].

**Conclusion:**

The risk of long-term opioid use after a bicycle trauma was low. However, opioids should be prescribed with caution, especially in those with injury to lower extremities or low educational level.

**Supplementary Information:**

The online version contains supplementary material available at 10.1007/s00068-022-02103-w.

## Background

The misuse of prescription opioids is a significant public health burden and is often described as an opioid crisis. In the general population of the United States (US), the use of prescription opioids is high. More than one third of US civilian adults reported to use prescription opioids in 2015 [1]. Orthopaedic trauma is one of the main reasons for patients to receive prescription opioids [2–4]. In Europe, opioids are not as frequently prescribed to trauma patients as in the US [3], and there is no evidence of a current or imminent opioid crisis [5, 6]. In recent years, however, the number of opioid prescriptions to non-cancer patients in Europe has increased [7, 8]. Little is known about the prescription of opioids in Sweden. During the last decade, opioids prescription in ambulatory patients in Sweden slightly decreased [9]. Simultaneously, the prescription pattern shifted from predominantly prescribed tramadol to oxycodone [9, 10]. Between 2006 and 2018, in parallel with the increase of oxycodone prescriptions from 3.17 to 30.33 per 1000, the oxycodone-related death rate increased from 0.10 to 1.12 per 100,000 [11]. It is unknown to what extent trauma contributes to the opioid prescription in Sweden. Recently, a Swedish study found an association between traumatic injury and long-term opioid use, as well as a higher mortality rate in these patients [12].

Long-term opioid use may lead to several problems, i.e., analgesic tolerance, opioid-induced hyperalgesia, physical dependency, or addiction [13–15]. Severe injury, injuries to the lower extremities, previous substance misuse, and psychiatric or somatic comorbidity have been identified as risk factors for long-term opioid use following trauma [12, 16, 17].

Trauma patients are a heterogenic patient group including both frail patients as well as young patients with substance misuse. In bicycle trauma patients, however, pre-existing comorbidities are less common than in the average trauma patients [18, 19]. Cyclists typically report excellent health prior to injury, are more likely to have an employment and a higher level of education compared to other patients injured in road traffic accidents [18, 19]. In Stockholm, like in other European cities, cycling is promoted by the authorities, and large investments in bicycle infrastructure are done. Consequently, the number of people using bicycles as a daily means of transport is increasing. Moreover, cyclists are vulnerable road users, and in all crashes, there is a risk of severe injury. Bicycle trauma patients are most frequently injured to the upper and lower extremities and are thereby at risk for long-term opioid use [16, 18, 20, 21].

The aim of this matched cohort study was to analyse the pattern of opioids prescription in a healthy trauma patient cohort, and whether bicycle trauma patients are at risk of becoming long-term opioid users. Further, the associations between injury to the lower extremities and low educational level and the risk of long-term opioid use were assessed.

## Methods

### Setting

This register-based cohort study was conducted at Karolinska University Hospital, Sweden’s largest Trauma Centre, covering an urban area of more than 2 million inhabitants. In Stockholm, all major trauma activity is centralised, and all patients suspected to be severely injured are referred to Karolinska University Hospital’s trauma unit (Karolinska Trauma Centre).

### Study cohort

The study cohort consists of all bicycle trauma patients registered in the Karolinska Trauma Register between January 2006 and December 2015. Non-Swedish residents were excluded. The study cohort consist of mainly non-electric pedal cyclists; e-scooters did not exist, and e-bikes still were very rare during the study-period. The Karolinska Trauma Register was launched in 2004 and includes all adult trauma patients (age ≥ 15) admitted to Karolinska University Hospital’s trauma unit, regardless of injury severity. In addition, all patients with Injury Severity Score (ISS) > 9 admitted to Karolinska University Hospital without activation of the trauma unit are also included in the register. Patients with isolated fractures of the upper or lower extremity, drowning, chronic subdural hematomas, mild burn injuries or hypothermia without concomitant trauma are not included in the Karolinska Trauma Register.

The annual number of bicycle trauma patients injured in road traffic accidents treated at Karolinska Trauma Centre is about 70, and a sample size of 700–900 bicycle trauma patients was anticipated [22].

### Matched comparators

The bicycle trauma patients were matched to comparators in a 1:5 ratio. The comparators were extracted from the Total Population Register which is managed by Statistics Sweden and collects microdata for demographic research and statistical purposes. The comparators were of the same age and sex and were residents in the same municipality at the same time as their respective index trauma patient. Furthermore, the comparators consist of individuals not found in the Karolinska Trauma Register.

### Data sources and definitions

From the Karolinska Trauma Register, information was retrieved on vital signs and Glasgow Coma Scale (GSC) at arrival to the hospital as well as injury pattern, injury severity, hospital length of stay and if the patients were admitted to the Intensive Care Unit (ICU). Overall injury severity was classified according to the ISS based on the Abbreviated Injury Scale (AIS) 1990 edition for year 2006, the AIS 2005 edition from 2007 and the AIS 2005 update 2008 version from 2011. Severe injury to a specific body region was defined as AIS score > 2.

Information on comorbidities and substance abuse for patients and comparators up to 8 years prior to trauma admission was obtained from the National Patient Register. The Swedish National Patient Register started in the 1960’s and is managed by the National Board of Health and Welfare (NBHW). It holds national cover for inpatient visits since 1987 and includes specialized outpatient care since 2001 [23]. Somatic comorbidity was defined as the presence of any of the somatic diagnoses included in the Charlson Comorbidity Index modified to ICD-10 [24]. Psychiatric comorbidity and substance abuse were defined as the presence of a diagnosis in ICD-10 groups F20-F99 and F10-F19, respectively.

Information on education was retrieved from the Longitudinal Integration Database for Health Insurance and Labour Market Studies (LISA), which started in 1991 and is managed by Statistics Sweden [25]. Level of education at the time of trauma was defined as low, medium, or high corresponding to ≤ 9 years, 10–12 years, and > 12 years of school attendance, respectively.

From the Prescribed Drug Register, information about dispensed opioid prescriptions was received from 6 months prior to the trauma until 18 months thereafter. The Swedish Prescribed Drug Register started in 2005 is managed by the NBHW and includes individual level data on dispensed prescription drugs in Sweden [26].

Pre-injury opioid use was defined as having received and dispensed at least one opioid prescription during the last 6 months before trauma. Long-term opioid use was defined as opioid use longer than 90 days [27]. In Sweden, drug prescriptions maximally cover three months. As a proxy for long-term opioid use, we used dispensed prescription opioids in the first quarter following trauma plus in at least one of the following quarters. Short-term opioid use was defined as dispensed prescription opioids in the first quarter after the trauma only, or in one of any other quarter after the trauma.

Equipotent doses of opioids were calculated using Oral Morphine Equivalents (OMEQ). In Sweden, methadone and buprenorphine are predominately used in opioid agonist therapy for individuals with problematic drug use and were therefore excluded (a list of included opioids and conversion rates is presented in Table 5 in the Appendix).

All-cause mortality, date, and cause of death were extracted from the Cause of Death Register which is managed by the NBHW. This register collects data on all deceased Swedish residents who died in Sweden or abroad and records cause of death as well as underlying and contributory factors [28].

### Statistical analysis

Categorical data were resented as proportions and percentages, continuous data as median and interquartile ranges. Categorical data were compared with the *χ*^2^ test, and continuous data with the Mann–Whitney *U* test.

Generalized Estimating Equations (GEE) regression models were used to analyse differences in mean opioid use among trauma patients and comparators before and after trauma. A *p* value < 0.05 was considered statistically significant; all tests were two tailed.

A directed acyclic graph (DAG) was used to help select potential confounding factors for the multivariable analyses, aiming to estimate effect of two selected exposures (educational level and injury to lower extremity) on the risk of long-term opioid use among injured bicycle trauma patients. For the logistic regression analyses, univariate and multivariable analyses were used to calculate the odds ratios (OR) with corresponding 95% confidence intervals (CI). In the multivariable analysis of educational level, adjustments for age (categories 15–44, 45–54, 55–64, 65–74, 75–84, ≥ 85 years), sex, somatic and psychiatric comorbidity were done. In the multivariable analysis of lower extremity injury, adjustments for age, sex, and for injury severity were done. Data analysis was performed using Stata/SE 14.2.

### Sensitivity analysis

By default, patients who received an opioid prescription in the first quarter after trauma and died during follow-up were classified as short-term opioid users. A sensitivity analysis was performed reclassifying these patients as long-term opioid users. An additional subset analysis was done including opioid-naïve patients only.

### Missing data

Data were analysed as complete cases.

## Results

We identified 907 bicycle trauma patients registered in the Karolinska Trauma Register between 2006 and 2015. These patients were matched with 4 535 comparators from the general population.

Compared with the comparators, bicycle trauma patients had a higher education level (*p* = 0.012) as well as a higher prevalence of psychiatric comorbidities and substance abuse (11.5% vs 7.9% and 7.5% vs 3.3%, respectively, *p* < 0.001). For somatic comorbidities, no difference was seen between trauma patients and comparators, whereas the 30-day as well as the 1-year mortality rates were significantly higher in the trauma patients than in the comparators (1.9% vs 0.1% and 2.8% vs 0.6%, respectively, *p* < 0.001) (Table 1).

**Table 1:** General characteristics for 907 bicycle trauma patients admitted to Karolinska Trauma Center in 2006–2015 and their matched comparators from the general population

	Trauma patients	Matched comparators	*p* value
Number	907	4535	
Age categories, *n* (%)			0.99
15–44	451 (49.7)	2225 (49.1)	
45–54	221 (24.4)	1095 (24.1)	
55–64	148 (16.3)	750 (16.5)	
65–74	62 (6.8)	330 (7.3)	
75–84	19 (2.1)	105 (2.3)	
≥ 85	6 (0.7)	30 (0.7)	
Age, median (IQR)	45 (31, 55)	45 (32, 55)	0.46
Male, *n* (%)	559 (61.6)	2790 (61.5)	0.95
Education level, *n* (%)			0.012
Low	134 (15.3)	854 (19.6)	
Medium	337 (38.6)	1631 (37.4)	
High	402 (46.0)	1871 (43.0)	
CCI categories, *n* (%)			0.95
CCI 0	796 (87.8)	3983 (87.8)	
CCI 1	57 (6.3)	292 (6.4)	
CCI > 1	54 (6.0)	260 (5.7)	
Psychiatric comorbidity, *n* (%)	104 (11.5)	375 (7.9)	< 0.001
Substance abuse, *n* (%)	68 (7.5)	150 (3.3)	< 0.001
Pre-traumatic opioid use, *n* (%)	35 (3.9)	228 (5.0)	0.13
Dead within 30 days	17 (1.9)	3 (0.1)	< 0.001
Dead within 360 days	25 (2.8)	25 (0.6)	< 0.001

Categorical parameters are presented as *n* (%), continuous parameters as median with interquartile range (IQR)

*CCI* Charlson Comorbidity Index

Most of the bicycle trauma patients suffered from a minor trauma (80.7%, ISS ≤ 15)*.* Hospitalization, however, was short with a median length of stay of one day (Table 2).

**Table 2:** Injury characteristics for 907 bicycle trauma patients admitted to Karolinska Trauma Center in 2006–2015

	Bicycle trauma patients
Number	907
ISS categories, *n* (%)	
≤ 15	732 (80.7)
> 15	175 (19.3)
ISS, median (IQR)	6 (4, 13)
Severe head injury*, *n* (%)	160 (17.6)
Severe thoracic injury*, *n* (%)	105 (11.6)
Severe abdominal injury*, *n* (%)	22 (2.4)
Severe spinal injury*, *n* (%)	36 (4.0)
Severe injury lower extremity*, *n* (%)	48 (5.3)
Severe injury upper extremity*, *n* (%)	5 (0.6)
Shock on arrival**, *n* (%)	13 (1.4)
GCS, *n* (%)	
13–15	810 (90.2)
9–12	33 (3.7)
3–8	55 (6.1)
ICU admission, *n* (%)	152 (16.8)
Hospital length of stay, median (IQR)	1 (1, 4)

Categorical parameters are presented as *n* (%), continuous parameters as median with interquartile range (IQR)

*ISS* Injury Severity Score, *GCS* Glasgow Coma Scale, *ICU* Intensive Care Unit

*Severe injury equal to Abbreviated Injury Scale (AIS) score > 2

**Shock on arrival equal to SAP (Systolic Arterial Pressure) < 90 mmHg on arrival to the trauma unit

After discharge from the hospital, 419 bicycle trauma patients (46%) received opioid prescriptions during the follow-up period, out of which 74 patients (8%) were classified as long-term opioid users. 488 bicycle trauma patients (54%) received no opioid prescriptions during the follow-up period. Data on education were missing in 3.7% of the patients (*n* = 34), all of them allocated in the reference group (short-term or no opioid users). In general, among bicycle trauma patients, long-term opioid users had a lower educational level (*p* = 0.006), a higher rate of pre-traumatic opioid use (18.9% vs 2.5%, *p* < 0.001) and were more likely to suffer from severe injury to the lower extremities than the short-term or no opioid users (16.2% vs 4.3%, *p* < 0.001) (Table 3).

**Table 3:** Characteristics for 907 bicycle trauma patients stratified on opioid use

	Reference group	Long-term opioid users	*p* value
Number	833	74	
Age, median (IQR)	44 (31, 55)	46 (39, 57)	0.15
Male, *n* (%)	516 (61.9)	43 (58.1)	0.52
Educational level, *n* (%)			0.006
Low	120 (15.0)	14 (18.9)	
Medium	298 (37.3)	39 (52.7)	
High	381 (47.7)	21 (28.4)	
CCI categories, *n* (%)			0.059
CCI 0	736 (88.4)	60 (81.1)	
CCI 1	52 (6.2)	5 (6.8)	
CCI > 1	45 (5.4)	9 (12.2)	
Psychiatric comorbidity, *n* (%)	92 (11.0)	12 (16.2)	0.18
Substance abuse, *n* (%)	62 (7.4)	6 (8.1)	0.84
Pre-traumatic opioid use, *n* (%)	21 (2.5)	14 (18.9)	< 0.001
ISS categories, *n* (%)			0.31
≤ 15	668 (80.3)	63 (85.1)	
> 15	164 (19.7)	11 (14.9)	
ISS, median (IQR)	6 (4, 13)	9 (5, 13)	
Severe head injury*, *n* (%)	156 (18.7)	4 (5.4)	0.004
Severe thoracic injury*, *n* (%)	95 (11.4)	10 (13.5)	0.59
Severe injury lower extremity*, *n* (%)	36 (4.3)	12 (16.2)	< 0.001
ICU admission, n (%)	146 (17.5)	6 (8.1)	0.038
Hospital length of stay, median (IQR)	1 (1, 4)	2 (1, 7)	0.001
Dead within 30 days	17 (2.0)	0 (0.0)	0.21
Dead within 360 days	24 (2.9)	1 (1.4)	0.44

Long-term opioid use was defined as prescription in the first quarter following trauma plus in at least one of the following quarters. The reference group were patients with short-term or no opioid use

Categorical parameters are presented as *n* (%), continuous parameters as median with interquartile range (IQR)

*CCI* Charlson Comorbidity Index, *ISS* Injury Severity Score

*Severe injury equal to Abbreviated Injury Scale (AIS) score > 2

Severe head injury was more common in patients with short-term or no opioid use (18.7% vs. 5.4%, *p* = 0.004). In a multivariable model, severe injury to lower extremity was associated with an increased risk of long-term opioids use [OR 4.88 (95% CI 2.34–10.15)], whereas high educational level was associated with a risk reduction [OR 0.42 (95% CI 0.20–0.88); Table 4].

**Table 4:** Univariate and multivariable logistic regression analyses, associations with long-term opioid use in bicycle trauma patients presented as OR (95% CI)

	Univariate analysis	*p* value	Multivariable model^§−§§^	*p* value
Severe injury to the lower extremity*	4.28 (2.12–8.65)	< 0.001	4.88 (2.34–10.15)^§^	< 0.001
Educational level				
Low	Ref	Ref	Ref	Ref
Medium	1.12 (0.59–2.14)	0.73	1.00 (0.51–1.96) ^§§^	0.995
High	0.47 (0.23–0.96)	0.038	0.42 (0.20–0.88) ^§§^	0.022

*Severe injury equal to Abbreviated Injury Scale (AIS) score > 2

^§^Adjusted for age categories, sex, and Injury Severity Score (ISS)

^§§^Adjusted for age categories, sex, somatic and psychiatric comorbidity

In a sensitivity analysis, excluding individuals with pre-traumatic opioid use (*n* = 35), the results for injury to lower extremities were unchanged [multivariable OR 5.45 (95% CI 2.52–11.80)], the multivariable OR for high education, however, was no longer significant [OR 0.49 (95% CI 0.21–1.12)].

**Fig. 1:**
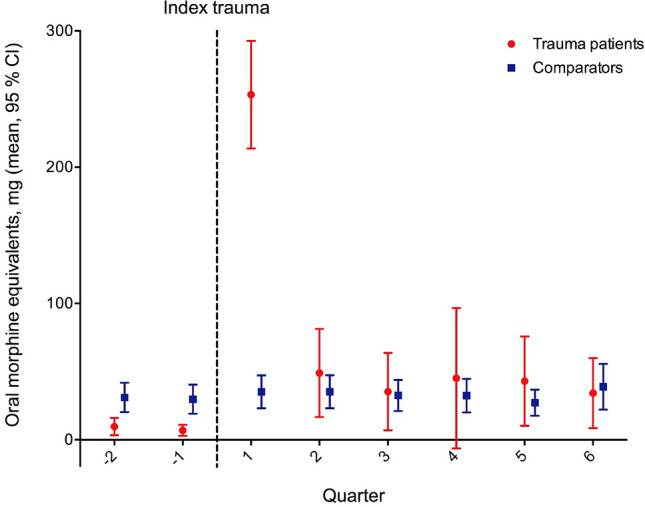
Opioid prescription before and after a bicycle trauma in 907 injured patients admitted to the Karolinska University Hospital in 2006–2015 and in 4535 comparators from the general population. Values show mean oral morphine equivalents (OMEQ) as milligrams per person per quarter for all trauma patients and controls. The dashed line indicates the time of the index trauma. 1 quarter equals 3 months

During the two quarters before trauma, the mean opioid consumption in bicycle trauma patients was significantly lower than in the comparators [− 21.3 mg (95% CI − 34.1 to − 9.0) and − 22.8 mg (95% CI − 34.5 to − 11.5)] (Table 6, Appendix); in the first quarter following trauma, the trauma patients mean opioid consumption peaked with 253.2 mg and fell thereafter to the same level as in the comparators (Fig. 1) (Table 6, Appendix).

Compared with their own pre-traumatic opioid use, bicycle trauma patients had an increased mean opioid use following injury. This increase was statistically significant for the first (245 mg, *p* < 0.001), second (41 mg, *p* = 0.013), fifth (35 mg, *p* = 0.036) and sixth (26 mg, *p* = 0.046) quarter post-trauma (Table 7, Appendix).

In a sensitivity analysis, patients who died during follow-up (*n* = 42) were reclassified as long-term opioid users. This did not alter the overall results in the logistic regression analyses. The protective effect of high education, however, did no longer reach statistical significance.

## Discussion

In this register-based cohort study originated from the largest trauma centre in Sweden, we analysed opioid prescription and risk of long-term opioid use in 907 bicycle trauma patients and their comparators. Prior to the trauma, the use of prescription opioids was lower in the bicycle trauma cohort compared with their comparators. In the first three months following the trauma, the use of prescription opioids was significantly higher in the bicycle trauma patients, but later conformed to the one of the comparators. Predictors for long-term opioid use were low-educational status, pre-traumatic opioid use, and severe injury to the lower extremities.

Most of the bicycle trauma patients were short-term opioid users or consumed no opioids at all. Only 8% of the trauma patients became long-term opioid users. Even though a lower number would be desirable, this percentage is comparable to the prevalence of prolonged opioid use in other studies [29–31]. Furthermore, some other studies reported an even higher prevalence of opioid use after musculoskeletal trauma at discharge from the hospital as well as in the longer term (54.3–97.1% and 19.7–35.3%, respectively) [32–35]. It is possible that the inconsistences are due to differences in national or local recommendations of opioid use and due to personal preferences of the prescribing physicians; some orthopaedic surgeons prefer opioids instead of non-steroidal anti-inflammatory drugs (NSAIDs) in concern about the risk of impaired fracture healing under NSAID treatment [36].

In line with previous studies, long-term opioid use was associated with low educational level, pre-traumatic opioid use, and injury to the lower extremities [12, 16, 17, 34, 37]. Injury to lower extremity was clearly associated with long-term opioid use, even though opioids are no first line treatment after orthopaedic trauma in Sweden. For acute non-cancer pain, the recommended standard medication is paracetamol in combination with NSAIDs. For severe pain, it is recommended to add opioids for a short-term treatment, according to the guidelines of the Centers for Disease Control and prevention, CDC [38, 39]. However, patients with injury to lower extremity seem to suffer more and longer standing from severe pain than patients with injuries to other body regions.

Regarding the low prevalence of somatic comorbidities and the low pre-traumatic opioid use in bicycle trauma patients, it is evident that these patients represent a healthy patient cohort. Furthermore, as the prevalence of somatic or psychiatric comorbidities was low in patients as well as in comparators, it is likely that the results are more generalizable to the general population than results from other groups of trauma patients. Surprisingly, psychiatric diagnoses and substance abuse were more common in the bicycle trauma group, although the educational level was slightly higher in the bicycle trauma patients. In the literature, low socioeconomic status is often associated with poorer mental health [40, 41]. A possible explanation to this different finding is that bicyclist might be more conscious about health issues than the general population, including mental health, and are more prone to seek help. Results from a Danish cohort-study showed, that in half of the individuals with disadvantageous socio-economic characteristics and poor mental health, the mental health problems were undiagnosed [42].

Despite this being a healthy cohort and established national recommendations on treatment of acute pain, there were new long-term opioid users in the cohort after the bicycle trauma.

The opioid crisis is most prevalent in the US, however, in recent years, the prescription of opioids to non-cancer patients has increased in Europe [7, 8]. It is well known that imprudent use of opioids may lead to analgesic tolerance, physical dependence, or addiction [13–15]. In this patient cohort from Sweden’s largest Trauma Centre, more than 50% of the patients were not in need of prescription opioids following the bicycle trauma. This evidence should encourage clinicians to adhere to pain management recommendations, and to prescribe opioids, if needed, only in the lowest effective dose for a very short time. In patients with expected long-standing pain, the optimal analgesic therapy should be multimodal, tailored to the individual, and with a responsible prescription of opioids, to reduce the risk of misuse and addiction [43–45]. This recommendation applies particularly to patients with prevalent risk factors, such as low educational level, injuries to the lower extremities and pre-traumatic opioid use.

The strengths of this study comprise its size, including all bicycle trauma patients admitted to Sweden’s largest Trauma Centre during a 10-year period. Beside the detailed information from the trauma register, additional information was retrieved from national registers for patients and comparators. In Sweden, a unique Personal Identification Number (PIN) is assigned to all Swedish residents at birth or at immigration to Sweden. This number is used in all contacts with e.g., health care, insurances, public authorities, or social services. The PIN facilitates linkage between different registers from different authorities. This enabled high-quality information on socioeconomic variables and comorbidity and ensured a minimal loss of follow-up. The rate of missing data was very low with less than 5%, which has been suggested as the maximum upper acceptance limit for large datasets [46].

The study is limited by its restriction to the Prescribed Drug Register. We only have information about dispensed prescription opioids; we do not know if the patients consumed the medication as prescribed. However, it is plausible that those who dispensed more than one prescription used the drugs corresponding to the first one. Moreover, we lack information on the indication of the opioid prescription. We can only assume that the prescriptions were connected to the index trauma. Furthermore, we only know the mean opioid consumption in oral morphine equivalents and have no information on type, dose, and potency of the opioids in each patient. This is a single-centre register-study from a large Trauma Centre, which may introduce a bias towards more severely injured patients. Thus, the opioid-use in our trauma cohort could be higher and longer standing than in cohorts from other hospitals. On the other hand, as patients with isolated fractures of the upper or lower extremities are not included in the Karolinska Trauma Register, we may have missed some patients at risk for long-term opioid use. Another limitation is that we lack detailed information about the circumstances leading to the accident, including the patient’s condition prior to the crash. Furthermore, as in all observational studies, there is a risk of residual confounding.

We suggest further research to focus on patients’ perspective and to evaluate the quality of the established pain management.

## Conclusion

In general, the risk of long-term opioid use after a bicycle trauma was low, as only 8% of the patients used opioids longer than 90 days. Severe injury to the lower extremities and low educational status were associated with an increased risk of long-term opioid use. Prescription of opioids to these patients should proceed with caution and under surveillance.

## Supplementary Information

Below is the link to the electronic supplementary material.

Supplementary file1 (DOCX 19 KB)
