# Supplementary material for: Long-term opioid use following bicycle trauma: a register-based cohort study
Source: Eur J Trauma Emerg Surg. 2022 Sep 12;49(1):531–8. doi: 10.1007/s00068-022-02103-w (PMC9925469; doi:10.1007/s00068-022-02103-w)
Supplement: Supplementary file 1 — Supplementary file1 (DOCX 19 KB) [file 68_2022_2103_MOESM1_ESM.docx]

**Supplementary Appendix**

**Table** **5.** Included ATC-codes and conversion rates for opioids. Anatomical Therapeutic Chemical (ATC) Classification System codes starting with N02A or R05DA0, Name, Route of administration, Defined Daily Dose (DDD), equianalgesic dose ratios and classification as strong or weak opioid.

| **ATC code** | **Name** | **Route** | **DDD (mg)** | **Equianalgesic dose ratio** | **Strong (S) or weak (W) opioid** |
| --- | --- | --- | --- | --- | --- |
| N02AA01 | Morphine | PO | 100 | 1 | S |
| N02AA03 | Hydromorphone | PO | 20 | 6 | S |
| N02AA05 | Oxicodone | PO | 75 | 1.5 | S |
| N02AA55 | Oxicodone combinations | PO | 75 | 1.5 | S |
| N02AB01 | Ketobemidone | PO | 50 | 1 | S |
| N02AB02 | Pethidine | PO | 400 | 0.1 | S |
| N02AB03 | Fentanyl | TD | 1.2 | 100 | S |
| N02AE | Buprenorphine | TD | 1.2 | 110 | S |
| N02AE | Buprenorphine | SL | 1.2 | 50 | S |
| N02AF | Nalbuphine |  |  | - | S |
| R05DA04 | Codeine |  |  | - | W |
| N02AX02 | Tramadol | PO | 300 | 0.2 | W |
| N02AX06 | Tapentadol | PO | 400 | 0.4 | W |
| N02AC04 | Dextropropoxyphene (chloride) | PO | 200 | 0.1 | W |
| N02AC04 | Dextropropoxyphene (napsylate) | PO | 300 | 0.1 | W |
| N02AC54 | Dextropropoxifen combinations excl psycholeptics | PO | 140 | 0.15 | W |
| N02AJ13 | Tramadol and paracetamol |  |  | - | W |
| N02AJ06 | Codeine and paracetamol |  |  | - | W |
| N02AJ08 | Codeine and ibuprofen |  |  | - | W |
| N02AJ09 | Codeine and other non-opioid analgesics |  |  | - | W |
| N02AA59 | Codeine combinations excl psycholeptics | PO | 100 | 0.1 | W |

PO = per oral, TD = transdermal, SL =sublingual, R = rectal, - = no filled prescriptions

**Table** **6.** Mean opioid consumption (mg OMEQ) for 907 bicycle trauma patients compared with 4 535 matched comparators, before and after the injury.

| **Calendar quarter before/after injury** | **Bicycle trauma patients mean consumption,**  **mg OMEQ** | **Comparators mean consumption, mg OMEQ** | **Mean difference (95% CI), mg OMEQ** | **P value** |
| --- | --- | --- | --- | --- |
| -2 | 9.7 | 31.2 | -21.3 (-34.1 – (-9.0)) | 0.001 |
| -1 | 6.9 | 29.9 | -22.8 (-34.5 – (-11.5)) | < 0.001 |
| 1 | 253.2 | 35.4 | 218.1 (176.6 – 259.1) | < 0.001 |
| 2 | 48.9 | 35.5 | 13.7 (-21.0 – 48.0) | 0.44 |
| 3 | 35.3 | 32.7 | 2.8 (-28.0 – 33.1) | 0.87 |
| 4 | 45.1 | 32.5 | 12.7 (-40.4 – 65.5) | 0.64 |
| 5 | 43.0 | 27.4 | 15.8 (-18.5 – 49.8) | 0.37 |
| 6 | 34.1 | 38.9 | -4.7 (-35.5 – 26.0) | 0.76 |

OMEQ, Oral Morphine Equivalents.

**Table 7.** Mean difference in opioid consumption (mg OMEQ) among bicycle trauma patients comparing each quarter after the injury with pre-traumatic opioid use (equal to average use during the 2 quarters preceding trauma).

| **Calendar quarter after trauma** | **Mean difference (95% CI), mg OMEQ** | **P value** |
| --- | --- | --- |
| 1 | 244.9 (205.8 – 284.1) | < 0.001 |
| 2 | 40.6 (8.6 – 72.7) | 0.013 |
| 3 | 27.0 (-1.3 – 55.3) | 0.062 |
| 4 | 36.8 (-14.5 – 88.1) | 0.16 |
| 5 | 34.7 (2.2 – 67.1) | 0.036 |
| 6 | 25.9 (0.40 – 51.4) | 0.046 |

OMEQ, Oral Morphine Equivalents.
